# CpG Island Methylation, Microsatellite Instability, and BRAF Mutations and Their Clinical Application in the Treatment of Colon Cancer

**DOI:** 10.1155/2012/359041

**Published:** 2012-06-26

**Authors:** Christina Wu, Tanios Bekaii-Saab

**Affiliations:** The Ohio State University Comprehensive Cancer Center, A454 Starling Loving Hall, 320 West 10th Avenue, Columbus, OH 43210, USA

## Abstract

There have been significant developments in colon cancer research over the last few years, enabling us to better characterize tumors individually and classifying them according to certain molecular or genetic features. Currently, we are able to use KRAS mutational status as a guide to therapy with anti-epidermal growth factor receptor antibodies. Other molecular features under research include BRAF mutation, microsatellite instability, and CpG island methylation. These three molecular features are often associated with tumors that have overlapping phenotypes and can be present simultaneously in the same tumor. However, they carry different prognostic and predictive qualities, making analysis of their interaction relatively complex. Much research thus far has examined the clinical relevance of microsatellite instability in helping determine prognosis and the predictive value of adjuvant 5-fluorouracil chemotherapy in stages II and III colon cancers. BRAF mutation appears to be a biomarker for poor prognosis. CpG island methylation is tightly associated with microsatellite instable tumors and BRAF mutation, but its clinical utility remains uncertain. Hereby, we examine preclinical and clinical data that supports the utilization of all three phenotypes in future research applied to clinical practice.

## 1. Introduction

Colorectal cancer (CRC) is the third most common cancer in the USA and the second leading cause of cancer death, with 141,210 annual new cases and 49,380 annual deaths in 2011 alone [1]. Survival for patients with CRC has improved dramatically over the last decade with the availability of 5-fluorouracil (5FU-) based doublet chemotherapy and the addition of molecularly targeted agents, such as, bevacizumab, cetuximab, and panitumumab in the metastatic setting [2–8]. Significant progress has been made in genotyping tumors and extracting clinically relevant information that may help guide us in early cancer detection, predictive biomarkers, and new target discovery.

CpG island methylation phenotype (CIMP), microsatellite instability, and BRAF mutation may have clinical significance in colon cancer. These characteristics are due to genetic and epigenetic changes and have overlapping histopathological features that often occur in the same tumor. Their interaction is complex; for example, microsatellite instability-high (MSI) tumors carry a good prognosis whereas the presence of a BRAF mutation confers a poor outcome [9–15]. In this paper, we describe the clinical significance of CIMP, microsatellite instability, and BRAF mutation and their potential interactions.

## 2. Microsatellite Instability

### 2.1. Background

Approximately 15% of colon cancers carries the MSI characteristic, and those cancers are histopathologically and clinically distinct from microsatellite instability-low and microsatellite stable (MSS) tumors [16, 17]. Microsatellite instability low and MSS tumors behave similarly biologically and are often classified as MSS collectively [18]. Microsatellite instability is defined as a change in the length of short repeating nucleotide sequences called microsatellites, and this instability is detected in tumor cells when compared to normal tissue [19].

Microsatellite instability is due to defects in the DNA mismatch repair (MMR) system, resulting in the accumulation of nucleotide mutations and alteration in microsatellite length [20]. The MMR system is responsible for the surveillance and repair of errors that occur during DNA synthesis. It is composed of 4 MMR proteins: MLH1, MSH2, MSH6, and PMS2 [21]. Germline mutation of the MMR proteins results in hereditary nonpolyposis CRC (HNPCC) otherwise known as Lynch syndrome, an autosomal dominant hereditary disease in which patients are prone to early development of colon cancer [21]. The majority of patients with MSI tumors have defects in the MMR system, which is caused by epigenetic gene silencing of hMLH1 by sporadic hypermethylation [22]. Due to the causal relationship of loss of MMR and resultant MSI tumors, immunohistochemical analysis of the MMR proteins may be used instead of MSI analysis. Absence of any MMR protein is accepted as representing a MSI phenotype. Thus, tumors are identified as proficient in MMR (pMMR) in presence of all MMR proteins or deficient in MMR (dMMR) if 1 or more of the proteins are absent [23]. A limitation of this test is that the loss of protein may be due to gene silencing by hypermethylation or germ line mutation. Therefore, further tests need to be performed to delineate the etiology of MMR deficiency.

Detection of microsatellite instability is by DNA extraction from either paraffin embedded or frozen tissue, followed by PCR amplification of specifically defined microsatellites [24]. PCR products are then analyzed by gel or capillary zone electrophoresis. The National Cancer Institute Workshop on Microsatellite Instability for Cancer Detection and Familial Predisposition compiled an accepted reference panel of 5 loci markers for identifying MSI: BAT25, BAT26, D5S346, D2S123, and D17S250 [25]. However, investigators have used >5 loci markers, with additional loci to the reference panel, for improved sensitivity. MSI is identified when >2 of the 5 loci are unstable, or when ≥30% of >5 loci are unstable [25].

### 2.2. Clinical Significance

Tumors with MSI are generally right sided and typically diagnosed at an earlier stage. Pathological features of MSI colon cancers include a Crohn's-like host response, mucinous phenotype, and high histological grade. Detection of MSI tumors in colon cancer is clinically significant, and retrospective studies of large randomized control trials in Stage II/III colon cancers have analyzed the prognostic and predictive value of MSI (Table 1) [9–12, 25, 26]. When examining the treatment arm in which no adjuvant therapy was given, the presence of MSI conferred a better prognosis than MSS. Interestingly, this positive prognostic effect seems more significant in stage III colon cancers, although more analysis is needed to validate these findings given the low incidence of MSI in colon cancers and the lower frequency of MSI tumors in stage III than stage II [26]. Tejpar et al. reported that in patients who did receive adjuvant chemotherapy, those with MSI tumors had superior overall survival (OS) and recurrence-free survival (RFS) compared to those with MSS tumors [12]. Moreover, patients with MSS tumors (especially Stage III cancers) seem to benefit the most from adjuvant 5-fluorouracil/Leucovorin (5FU) chemotherapy [9, 10]. Conversely, patients with MSI tumors and more specifically those with stage II cancer, do not seem to benefit from adjuvant chemotherapy [9, 10]. Overall, the absolute risk reduction with adjuvant 5FU therapy in stage II colon cancer is low. Practicing oncologists typically refer to clinicopathologic high-risk features (e.g., T4 lesion, bowel obstruction, lymphovascular invasion, perineural invasion, perforation, positive margins, or <12 lymph nodes sampled at surgery) to determine whether adjuvant therapy should be offered. We believe that based on the published data, tumor MSI status should be integrated into clinical decisions about therapy for stage II colon cancer [9–12, 25, 26].

Patients with stage III colon cancer did not have differences in benefit from adjuvant 5FU treatment when comparing tumors with MSI versus MSS [11]. However, results from a recent study suggest that patients with stage III MSI tumors, and more specifically those with germ line mutation, seem to benefit from adjuvant 5FU chemotherapy [26]. It is unknown how these results apply to the current standard adjuvant therapy for stage III colon cancer which consists of adding oxaliplatin to 5FU, suggesting the need for further study.

One of the major clinical implications of finding MSI tumors is that it is a cost-effective method to screen for HNPCC. Once dMMR tumors are detected, they can then be tested for the germ-line mutation of the missing MMR protein. Identifying HNPCC can lead to a great impact on survival outcomes for patients and their families through early introduction of genetic counseling and cancer screening practices. In the CAPP2 study, 861 HNPCC patients were randomized to receive aspirin or placebo for 2 years, and the treatment arm was found to have a reduced incidence of cancer with a HR of 0.41 (0.19–0.86, *P* = 0.02) [41]. As such, identifying those at risk may help with primary prevention for colon cancer in this patient population.

Preclinical studies have shown increased sensitivity of MSI cancer cell lines to irinotecan, which should be explored in the clinical setting [42]. Although irinotecan has not shown to be effective in the adjuvant setting in stage III cancers, it may prove to be effective in MSI tumors specifically [43]. A recent study reported that patients with stage III colon cancer who had MSI tumors and were treated with 5FU and irinotecan (IFL) had an improved 5-year disease-free survival (DFS) when compared to MSS tumors with HR of 0.76 (95% CI, 0.64 to 0.88) versus 0.59 (95% CI, 0.53 to 0.64), respectively (*P* = 0.03) [11]. In contrast, another study of 1,327 patients with stages II and III cancers, infusional 5FU, and irinotecan did not provide additional benefit in OS or RFS over 5FU treatment alone for MSS or MSI tumors [12]. More work, however, needs to be done to explore the role of irinotecan in the adjuvant setting. Finally, preclinical data suggests a role for poly (ADP ribose) polymerase (PARP) inhibitors as candidates to target MSI tumors via synthetic lethality [44]. PARP inhibitors block base excision repair and cause double-stranded DNA breaks, which MSI cells are unable to repair thus causing tumor cell death [44]. This strategy has already been employed in the treatment of BRCA mutant breast cancer [45]. Currently clinical trials are ongoing examining the efficacy of PARP inhibitors in MSI metastatic colon cancers.

## 3. CpG Island Methylation Phenotype (CIMP)

### 3.1. Background

CIMP is detected in approximately 30–40% colon cancers [46, 47]. CpG island methylation is DNA methylation at the cytosine base of CpG dinucleotide islands, by DNA methyltransferase enzymes [48]. The normal genome contains about 70–80% CpGs that are usually methylated, but CpG islands located proximal to the promoter region of genes are usually left unmethylated [49]. Conversely, in cancer cells it has been observed that there is genome-wide hypomethylation and gene promoter hypermethylation. Hypermethylation contributes to gene silencing and genomic instability and affects tumor-suppressor genes, DNA repair, and cell-cycle control.

Methylation analysis is performed by either methylation-specific polymerase chain reaction (PCR) or real-time PCR (Methylight) [50, 51]. One of the challenges in studying CIMP tumors is that there is no general consensus of which specific methylated loci to use to define CIMP. The majority of studies have typically included the classic panel: hMLH1, p16, MINT1, MINT2, and MINT31 [27]. However, in addition to these 5 loci, CIMP marker panel may be extended to include: CACNA1G, CRABP1, IGF2, NEUROG1, RUNX3, SOCS1, HIC1, IGFBP3, and WRN, with no consensus on how many markers are required to be positive to define CIMP [27–36, 38–40]. When analyzing clinical outcome results across studies, caution should be taken because the loci marker panel and criteria for CIMP vary.

Toyota et al. first described CIMP in CRC, identifying cancer-specific methylation and distinguishing it from age-specific methylation [48]. Subsequently, Weisenberger et al. performed unsupervised two-dimensional cluster analysis of DNA methylation and classified CRC into CIMP-negative or CIMP-positive cancers [52]. They observed a strong relationship of CIMP cancers with BRAF mutations. In addition, there is now evidence of different subgroups of CIMP (high, low, and negative) [53]. In an analysis of 97 colon cancer tumors, whereby genetic and epigenetic alterations were studied, unsupervised hierarchical clustering of DNA methylation identified 3 distinct groups of CIMP, described as CIMP1, CIMP2, and CIMP-negative [54]. CIMP1 tumors were highly associated with MSI (80%) and BRAF mutation (53%). CIMP2 tumors had a high incidence of KRAS mutations (92%), and CIMP-negative correlated with p53 mutations (71%). A group from Singapore performed comprehensive methylation profiling of 1,505 CpG sites and identified 3 distinct CIMP groups (CIMP-H, CIMP-M, and CIMP-L), with different features to those described in the previous study, for example, CIMP-H cluster has a high incidence of KRAS and BRAF mutation and thus now suggests 4 CIMP categories [55]. CIMP classification continues to be refined as technology improves.

### 3.2. Clinical Significance

Pathological features of CIMP tumors are the high rate of mutations (KRAS or BRAF), wild-type p53, proximal colon location, and higher occurrence in women and older patients. Characterizing CIMP alone is not routinely performed to guide treatment decisions. It is currently being explored as a marker for genetic and environmental factors that affect colon carcinogenesis and as a possible prognostic or predictive marker along with MSI and BRAF mutation. Interestingly, there has been an association reported between smoking and CIMP. In smokers, there is increased CpG methylation at the bronchial epithelium [56]. In colon cancer, cigarette smoking is associated with CIMP tumors and has a significant relationship to the number of cigarettes smoked [57]. Also, the increased risk for colon cancer in inflammatory bowel disease (IBD) is hypothesized to be due to repetitive mucosal inflammation and possibly age-related CIMP [58]. However, studies have not showed an increased incidence of CIMP in IBD-associated CRC as compared to sporadic cancers [59, 60]. A study from Cleveland Clinic matched 19 patients who had CRC and ulcerative colitis to 54 patients with sporadic CRC, and their tissue was examined for BRAF mutation, CIMP, KRAS mutation, and p53. The colitis-associated CRC did not have an increased number of patients with CIMP (5% versus 22%) but was more likely to have a p53 mutation (95% versus 53%, *P* = 0.001) [54]. Similarly, a methylation microarray analysis of IBD CRC, sporadic CRC, and normal colonic tissue demonstrated that CIMP was less common in IBD-associated CRC than sporadic CRC [55]. Thus, it is unlikely that IBD-related CRC is via the CIMP pathway.

The predictive role of CIMP is controversial. It has been hypothesized that CIMP tumors have aberrant folate metabolism in cancer cells and thus could be more sensitive to antifolate therapies, such as 5FU [61, 62]. Conversely, adjuvant 5FU-based therapy is not beneficial in stage II colon cancers that are identified as MSI, and there is overlap between MSI and CIMP tumors, thus CIMP presence was also postulated to be a negative predictive marker for 5FU therapy. There are studies in which the presence of CIMP has predicted benefit of 5FU-based treatment in stages II/III colon cancer; however, there is also evidence showing a trend for resistance to chemotherapy in CIMP tumors (Table 2) [27, 28, 30, 31, 38, 39]. In the majority of the studies that have analyzed patients with all stages of CRC who did not receive chemotherapy treatment, tumors identified as MSS, and CIMP have a worse survival outcome [27, 29–34, 36]. However, two studies report better outcomes with CIMP tumors [35, 39]. The conflicting data may be due to the different criteria used across the studies to define CIMP status, or that CIMP tumors are heterogenous and need to be further classified. Based on the data available, CIMP cannot be used clinically as a prognostic or predictive marker.

DNA hypermethylation is under investigation both as a tool in colon cancer screening and a target for cancer therapy. One method of utilizing DNA methylation in CRC screening is by detecting abnormal DNA methylation from tumor cells shed in stool samples. In one study, stool samples were collected from patients who had undergone endoscopic examination and biopsy and identified as healthy control patients and patients diagnosed with adenomas or CRC [63]. DNA methylation was analyzed by Methylight. For diagnosing CRC, sensitivity was 90% (CI 56–100%) and specificity was 77% (CI 46–95%). Another proposed method to detect aberrant DNA methylation is by testing for cell-free DNA that is released by cancer cells undergoing apoptosis or necrosis in the serum or plasma [64]. However, a number of obstacles to this method exist including extracting sufficient DNA from the sample and determining which platform assays to use.

Hypermethylated CpG islands are also targeted in drug development. Preclinical studies suggest that azacytidine and decitabine, both DNA methyltransferase inhibitors, are able to demethylate CpG islands in colon cancer cell lines in a nonrandom and reproducible fashion [65]. There is currently an ongoing clinical trial with azacytidine in combination with entinostat, a histone deacetylase inhibitor in the treatment of advanced colon cancer. Another phase I/II trial studies the combination of azacitidine, capecitabine, and oxaliplatin in advanced CRC, with specific selection for patients with CIMP tumors.

## 4. BRAF

### 4.1. Background

The incidence of BRAF mutation V600E in colorectal cancer is 8–10% [66]. BRAF mutation is detected by DNA extraction from paraffin-embedded or frozen tissue and then analyzed by PCR amplification and either pyrosequencing or Sanger sequencing. The RAS/RAF/MAPK pathway is important in tumor cell proliferation, invasion, and inhibition of apoptosis [67, 68]. Activation of the MAPK pathway is initiated by ligand binding to a receptor tyrosine kinase, such as, epidermal growth factor receptor (EGFR) that has been shown to be critical in the development of colorectal cancer. The RAS proteins are small GTPases that are farnesylated and inserted into the cell membrane. They are activated downstream of EGFR and propagate signaling by recruiting BRAF to the plasma membrane. BRAF in turn phosphorylates MEK1/2. Once activated, MEK1/2 phosphorylates ERK1/2 which subsequently triggers downstream signaling by phosphorylating cytoplasmic and nuclear transcription factors and proteins. Both KRAS and BRAF mutations are mutually exclusive and may indicate that they individually play an important role in the MAPK pathway. BRAF mutation is commonly present in cancers, especially melanoma and colon cancers [69]. Eighty percent of the mutations is attributed to the V600E substitution in the kinase domain. Mutant BRAF results in increased serine threonine kinase activity, and activation of the RAS/RAF/MAPK pathway [70]. *In vitro* studies have shown that BRAF mutation correlates with ERK 1/2 activation in CRC lines [71]. BRAF mutants elicit tumorigenic properties, as demonstrated when transfection of BRAF mutant proteins transformed NIH3T3 cells and BRAF mutant expressing NCM640 cells underwent malignant transformation by gaining the ability to grow on soft agar [70, 72]. BRAF mutation also affects the mitotic spindle and spindle assembly [73, 74].

### 4.2. Clinical Significance

It is recognized that KRAS mutations in codon 12 and perhaps 13 are predictive of lack of efficacy of anti-EGFR monoclonal antibody therapy in advanced CRC [7, 8, 75, 76]. Studies have examined whether mutations in BRAF, which is downstream of KRAS, may also have an impact on the efficacy of anti-EGFR agents such as; cetuximab or panitumumab. One retrospective study suggested that the presence of BRAF mutation may be an adverse predictive biomarker for the activity of cetuximab in MCRC [60]. Another retrospective analysis of 113 patients who received panitumumab or cetuximab showed that none of the patients with a BRAF mutation responded to treatment [77]. However, retrospective analysis of the large phase III CRYSTAL trial in which patients with metastatic CRC were randomized to 5FU and irinotecan with or without cetuximab showed that patients with BRAF mutant tumors did poorly, regardless of the therapy they received [13]. This finding was also confirmed in the MRC FOCUS phase III trial [78]. Additionally, evaluation of BRAF in stages II and III colon cancers showed that BRAF mutation was a negative prognostic factor for overall survival in patients [15, 37, 40]. Compilation of all this data indicates that the presence of a BRAF mutation is prognostic rather than predictive.

BRAF mutation analysis is not yet routinely tested in the clinical setting. Nonetheless, BRAF mutation is being studied as a target for various therapeutic agents. In BRAF-mutated CRC, a number of novel agents are being developed to target the RAS/RAF/MAPK pathway, including BRAF inhibitors and MEK inhibitors. The selective BRAF inhibitor, PLX4720, has been shown to inhibit MAPK phosphorylation in BRAF mutated cancer cell lines [79]. PLX4720 causes cell-cycle arrest, apoptosis, and tumor growth delays in BRAF mutant tumor xenograft models. There is an ongoing trial of the BRAF inhibitor, PLX3603, in BRAF mutant cancers. Vemurafenib, a FDA-approved BRAF inhibitor for the treatment of BRAF-mutant melanoma, has only had a modest response rate in CRC [80]. A RNA-interference-based genetic screen was utilized in cancer cells treated with vemurafenib, to explore whether the knockdown of selective kinases would synergize with vemurafenib [81]. Interestingly, EGFR blockade and BRAF mutation inhibition had strong synergy. Despite inhibition of BRAF mutation there was continued proliferation due to the feedback activation of EGFR. A clinical trial testing the combination of BRAF inhibition and anti-EGFR therapy would be indicated in patients with EGFR expressing and BRAF mutant tumors. BRAF mutation is also indicative of MAPK pathway activation, and thus MEK inhibition is another targeted therapy that has been under investigation [71]. In BRAF/KRAS mutant CRC cell lines, the MEK inhibitor, CI-1040, has been shown to impair anchorage-independent growth [71]. There is an ongoing phase I/II trial of selumetinib, a MEK inhibitor, in combination with irinotecan in KRAS and BRAF mutant CRC. Also, there are trials with single-agent MEK162 or AZD6244, both MEK inhibitors in BRAF mutant cancers. Regorafenib is a multikinase inhibitor that was recently reported to have improved survival outcomes versus placebo in patients with metastatic CRC (although patients were not selected for BRAF mutation) [82].

## 5. Potential Interactions between MSI and the Presence of CIMP and BRAF Mutation

There is considerable overlap among the tumors characterized as MSI, CIMP, and BRAF mutant [53]. Tumors that harbor BRAF mutation, MSI, and CIMP are generally proximal and mucinous tumors [83]. In a population-based study of 1315 patients with colon cancer, cigarette smokers had a higher incidence of MSI, CIMP, and BRAF mutant tumors [58]. Cigarette smoking was associated with CIMP and BRAF mutant tumors, odds ratio (OR) 2.85 (95% CI 1.53–5.29), and MSI cancer OR 3.43 (95% CI 1.57–7.50). This suggests that genetic mutation and epigenetic alterations due to environmental insults could concomitantly contribute to carcinogenesis. When Weisenberger et al. first classified CRC tumors as CIMP positive and CIMP negative, they observed that all BRAF mutant tumors were CIMP positive and that sporadic MSI cancers had CIMP methylation of *MLH1* [52]. The presence of all three features suggests that they contribute to colon carcinogenesis. Sessile serrated polyps, which account for 40% of colon cancers, are precursor lesions that lead to CIMP carcinoma and Lynch syndrome cancers [84]. BRAF mutation is likely the activating mutation in the serrated pathway, as it is commonly present in serrated adenomas and inhibits normal apoptosis of colon cells [71, 85, 86]. In turn, the serrated adenomas are prone to methylation, especially the *MLH1* gene. In a study that assayed 79 sporadic polyps, it was demonstrated that as histological changes were more advanced, from polyp to carcinoma, there was a correlation with increased methylation levels [87]. Also, to support that BRAF mutation is the first initiating step in carcinogenesis, *in vitro* studies have demonstrated that that transfection of BRAF mutant plasmids into CaCO2 cells induced MSI, resulting in a lower DNA content, cell cycle distribution, and downregulation of DNA repair genes [88]. However, transfecting CIMP-negative, BRAF wild-type colorectal cell lines with BRAF mutant expressing vector was not sufficient to cause DNA hypermethylation [89].

Serrated pathway syndrome is a familial colorectal cancer that is predominantly BRAF mutant, in contrast to Lynch syndrome. In the initial report, 43 individuals from 11 families were studied and all patients met Bethesda guidelines [90]. There were BRAF mutations in 63% polyps and 70% cancers. Eighty percent of the patients had *MINT31 *hypermethylation and MSI status was variable, which suggested a differing carcinogenesis pathway than sporadic MSI tumors which are BRAF mutant and CIMP (with hypermethylation at *MLH1*). Their polyps were serrated in description and the cancers were early in onset. A separate study screened 194 colorectal tumors from patients with family history of colon cancer [91]. BRAF mutation was identified in 100% (*n* = 8) MSI tumors, and BRAF mutation correlated with extracolonic tumors among the families. BRAF mutation may be the initiating step in carcinogenesis for MSI tumors that are not due to HNPCC, in both sporadic and familial CRC, and may warrant specific targeting in this patient population in future trials.

BRAF mutation occurs at a higher frequency in CIMP tumors than non-CIMP tumors and can be used as a surrogate marker for CIMP in many instances. One study examining CIMP and BRAF-mutation in 460 tumors showed that CIMP occurred at a higher frequency in BRAF mutated tumors (68%) than BRAF wild-type tumors (5.2–12%) [92]. BRAF mutations are also very common in MSI tumors (50%) but rarely occur in HNPCC tumors [66, 93]. The evaluation of MSI and BRAF mutation together is a useful tool for screening HNPCC in the colon cancer population. For example, all patients who have colon cancer are screened for HNPCC in our institution with the following algorithm: samples are tested for the presence of MMR proteins by immunohistochemistry. Recognizing that the absence of MMR proteins could be due to either epigenetic methylation or germ line mutation, we then proceed with the detection of BRAF mutations as a surrogate for CIMP or *hMLH1 *methylation. If the tumor is BRAF wild type, we then perform gene mutation analysis to separate the tumors with germ line mutation from tumors that have MSI phenotype due to hMLH1 methylation and are BRAF wild type.

Although CIMP, MSI, and BRAF mutation may be present in the same tumor, there are also clearly distinct clinical outcomes observed across the 3 categories (Table 2). MSI is associated with a better prognosis for patients with stage II and III CRC and in contrast BRAF mutation is associated with worse prognosis. It is unclear what that may imply on tumors that are MSI and BRAF mutant. Likewise, it is uncertain what level of interaction exists between CIMP and BRAF mutation when they tend to be present together in a number of tumors. In a study of 649 colon cancers, patients with MSI tumors had superior cancer-specific mortality when compared to MSS tumors in univariate analysis, HR 0.38 (95% CI 0.22–0.66). However, in the multivariate analysis accounting for MSI, CIMP, and BRAF mutation, this advantage was attenuated [35]. BRAF mutation was a marker for poorer prognosis, and no protective effect was seen with MSI versus MSS in BRAF mutant tumors. Patients with CIMP high tumors fared better than CIMP low tumors regardless of MSI status or BRAF mutation status. However, the majority of studies have identified CIMP as a marker for worse outcome (Table 2).

## 6. Conclusion and Future Directions

Studies examining interactions between MSI and the presence of CIMP and BRAF mutation and their clinical outcomes are summarized in Table 2. However, due to the retrospective nature of the majority of the studies, dependence on tissue availability, and low incidence of BRAF mutations and MSI tumors, the disease stages are not matched with treatment and clinical outcome. Nonetheless, a few broad conclusions can be made. MSI tumors consistently predict for better clinical outcome than MSS tumors. BRAF mutation is a poor prognostic marker; however, its interaction with MSI has to be further clarified. Patients with MSS tumors that harbored BRAF mutation or CIMP fared worse. It is interesting that one study reported CIMP to be associated with better patient outcomes, which is contrary to the other reported studies [35]. This could be due to different panel markers that they used to define CIMP or due to the different patient population in their study. Whether CIMP tumors respond or are resistant to 5FU-based chemotherapy is confounding as well and likely points to the heterogeneity of the tumors studied, due to CIMP criteria and differing disease stages. Clearly, there is a great paucity of information in the analysis of the tumor-subtype interactions.

Further information needs to be gleaned from these distinct but occasionally overlapping tumor subtypes, with regards to clinical outcomes in the different disease stages and sensitivity to various therapies. In stage II disease, oncologists already utilize MSI status to help guide their decision in adjuvant 5FU therapy. Other prognostic but necessarily predictive tools that are being developed for stage II cancer include Oncotype DX, a 12-gene assay using real-time reverse-transcriptase PCR, which examines 7 cancer genes and 5 reference genes to determine a recurrence score. This technology has been validated with the QUASAR trial, a phase III trial of stage II colon cancer patients who were randomized to adjuvant 5FU or observation alone [94]. Characterizing tumors further by BRAF mutation and CIMP status could potentially dissect out the differences the tumors may have and be used as prognostic and predictive biomarkers. We have already begun utilizing MSI and BRAF mutational status in a systematic way to screen for HNPCC in patients with colon cancer. However, investigating the different behavior of MSI tumors that are the result of germ line mutation versus hypermethylation has not been established, largely due to small numbers of HNPCC patients. There are likely significant differences that could alter the way we treat our patients. Additionally, it is essential to have a consensus on a standardized panel of loci to define CIMP similar to the standardized panel utilized to identify MSI.

In conclusion, personalized medicine has become a significant part of the modern management of colon cancer. One example is the selection of patients who are more likely to respond to anti-EGFR therapy by excluding KRAS mutant tumors. Based on the current data available, our recommendations are for all patients with CRC to be tested for MSI status. If the tumor is dMMR, then follow-up tests including BRAF mutation and genetic testing will identify patients with HNPCC. Also, for stage II colon cancer patients, the presence of dMMR along with favorable histopathological features would justify no adjuvant chemotherapy. Patients with mCRC should have their tumor checked for BRAF mutation if they do not have KRAS mutation, to see if they are eligible for clinical trials for BRAF mutation inhibitors or drugs targeting the MAPK pathway. Routine CIMP testing is not recommended. Further unraveling of the CIMP, BRAF, and MSI findings will enable us to target selected patients, identify their recurrence risk, and make decisions about their treatment options across all stages of colon cancer.

## Figures and Tables

**Table 1:** Retrospective studies of randomized control trials that examined efficacy of adjuvant 5FU chemotherapy in patients with MSI versus MSS colon cancers.

Reference	No. of patients	Stage	MSI(%)	Adjuvant treatment	Outcome		
					Better		Worse
[9]^#^	570	II/III	17	No treatment	MSI		MSS
				5FU or 5FU/levamisole	MSS, more benefit from therapy		MSI
[25]	542	II/III	18	No treatment	MSI = MSS		
				5FU	MSI = MSS		
[11]	792	III	13	5FU	MSI = MSS		
				5FU + irinotecan	MSI, trend for better outcome than 5FU alone		MSS
[12]	1,327	II/III	14	5FU	MSI		MSS
				5FU + irinotecan	No added benefit with irinotecan MSI = MSS		
[10]^#^	1027	II/III	16	No treatment	MSI		MSS
				5FU or 5FU/levimisole	MSS and Stage III—benefit with treatment		MSI and stage II-no benefit with treatment
[26]^#^	2141	II/III	16	No treatment	MSI		MSS
				5FU-based therapy $\hat{\;}$	MSI and stage III-benefit with treatment*		

5FU = 5FU + leucovorin

^#^Data pooled from overlapping trials.

$\hat{\;}$5FU in addition to: levamisole, portal venous 5FU, interferon-gamma, immunotherapy, vincristine, or semustine.

*Most treatment benefit was in patients with MSI tumors due to germ line mutations.

**Table 2:** Studies that examined interactions of MSI, CIMP, and BRAF mutation in colon cancer.

Reference	No. of patients	Stage	MSI (%)	CIMP (%)	BRAF mt (%)	Outcome (no treatment)		5FU-based treatment
						Better	Worse	
[27]	605	I–IV	12	16		MSI	MSS/CIMP	MSI and CIMP did not predict outcome with therapy
[28]	206	III	14	33				CIMP predicted benefit with therapy
[29]	911	I–IV	9	27	9.5	MSI/BRAF mt or wt	MSS/BRAF mt MSS/CIMP	
[30]	30	IV	0	10			CIMP	CIMP showed trend for resistance to chemotherapy, NS
[31]	188	IV		15			CIMP	CIMP did not predict outcome with therapy
[32]	582	I–IV	14	17	13	MSI	MSS/CIMP	
[33]	134	II-III	31	14	5		MSS/CIMP MSS/BRAF mt	
[34]	130	I–IV	15	18			MSS/CIMP	
[35]	649	I–IV	19	19		CIMP, MSI	BRAF mt	
[36]	604	I–IV	6	8	15		MSS/CIMP	
[37]	1,913 (MMR) 1,584 (BRAF)	II	11		8	MSI	MSS	MSI and BRAF mt did not predict outcome with therapy
[38]	245	I–IV	20	14	5			Stages II/III patients with CIMP benefit from adjuvant treatment
[39]	302	I–IV	25.8	32.7	21	CIMP		CIMP tumor did not benefit from adjuvant treatment
[40]	506	III	15		15	MSI/BRAF wt	MSS/BRAF mt	MSI/BRAF wt and MSS/BRAF mt tumors showed a trend for benefit from IFL, NS

BRAF mt: BRAF mutation, BRAF wt: BRAF wild-type, NS: not statistically significant, IFL: 5FU, leucovorin, and irinotecan.
